# The Importance of Long Time Follow-Up after Vital Pulp Therapy: A Case Report

**Published:** 2008-07-10

**Authors:** Parviz Amini, Masoud Parirokh

**Affiliations:** 1 *Department of Prosthodontics, Oral and Dental Diseases Research Center, School of Dentistry, Kerman University of Medical Sciences, Kerman, Iran*; 2 *Department of Endodontics, Oral and Dental Diseases Research Center, School of Dentistry, Kerman University of Medical Sciences, Kerman/ Iranian Center for Endodontic Research, Tehran, Iran*

**Keywords:** Calcium Hydroxide, Follow-Up, Open Apex, Vital Pulp Therapy

## Abstract

This report describes a case of an eight years old girl who was treated for complicated crown fracture of right maxillary central incisor because of a sport accident. For the tooth total pulpotomy was performed in order to achieve apexogenesis and the tooth was restored with a composite resin. The patient was reviewed over 10 years. At first the tooth showed continued root development and complete apex formation following vital pulp therapy, however, after 10 years the tooth developed pulp necrosis and periapical radiolucency. Following root canal therapy, periapical radiolucency has been healed.

## INTRODUCTION

Vital pulp therapy is the treatment of choice for the teeth with vital pulp and open apexes following mechanical or carious pulp exposure (1,2). The procedure is performed with amputating coronal pulp and covering the rest of the vital pulp with capping materials such as calcium hydroxide (CH) or mineral trioxide aggregate (MTA) (3).

Some investigators believe that following pulp development and apex closure there would be no need for root canal therapy (4), in contrast to many others who are emphasizing root canal therapy following complete apical foramen formation (5). Several reports and investigations showed successful treatment of pulp exposure of the teeth with open apexes (6-10). An investigation on long-term prognosis of complicated crown fracture reported no pulp necrosis up to 17 years after the injury (11). This case report represents a case that was followed- up for 10 years and showed pulp necrosis following early successful root development.

## CASE REPORT

**Figure 1:**
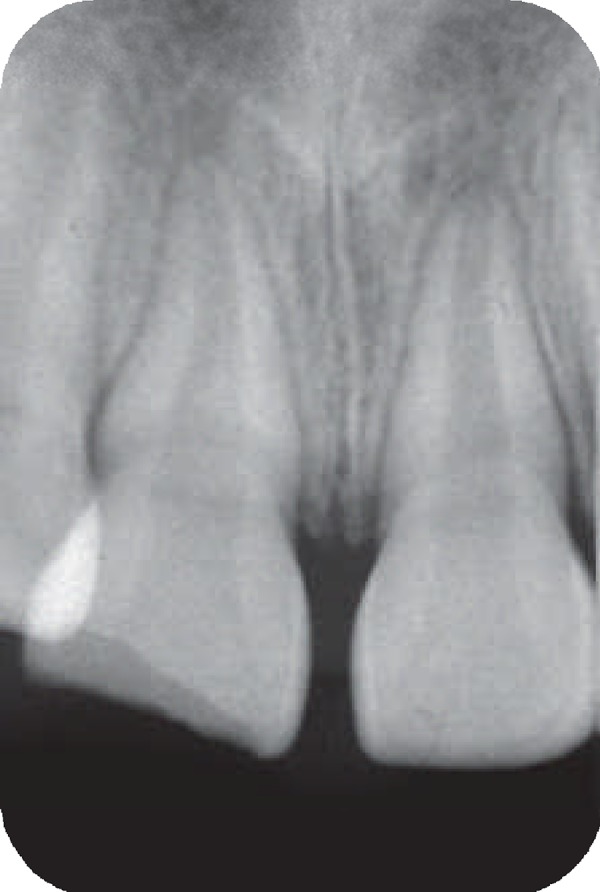
Image of the right maxillary central incisor before treatment

**Figure 2:**
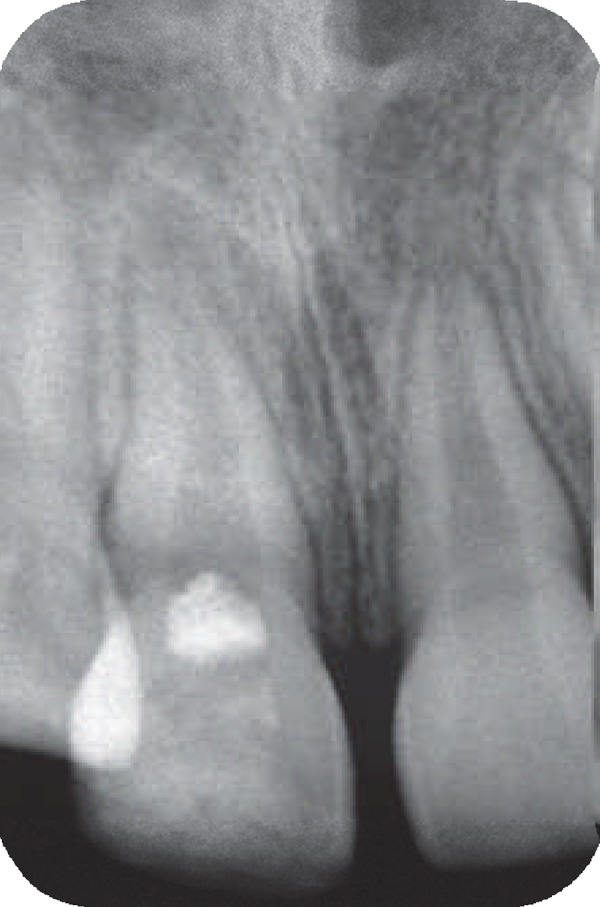
Radiographic image of the same tooth 6 months after treatment

**Figure 3:**
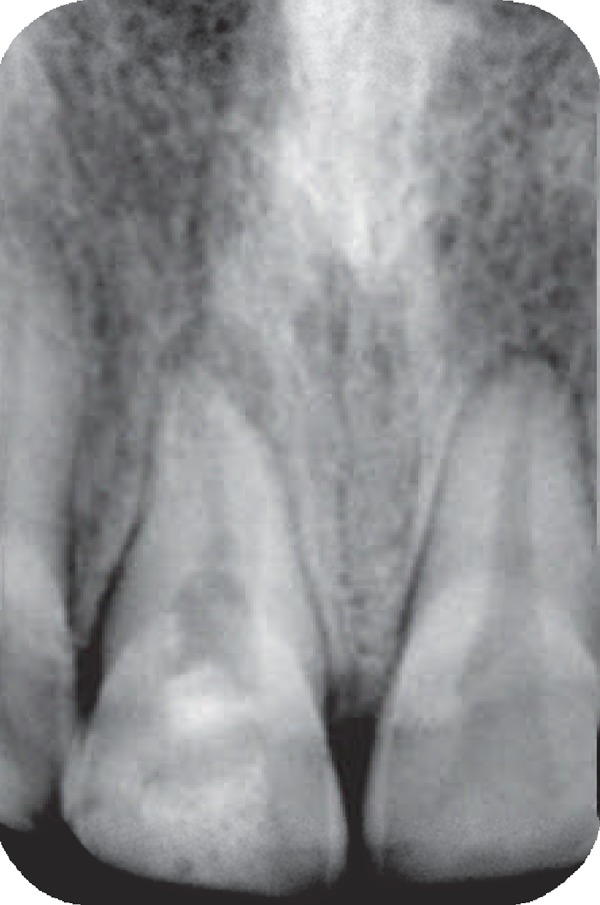
Presence of apical radiolucency after 10 years

**Figure 4:**
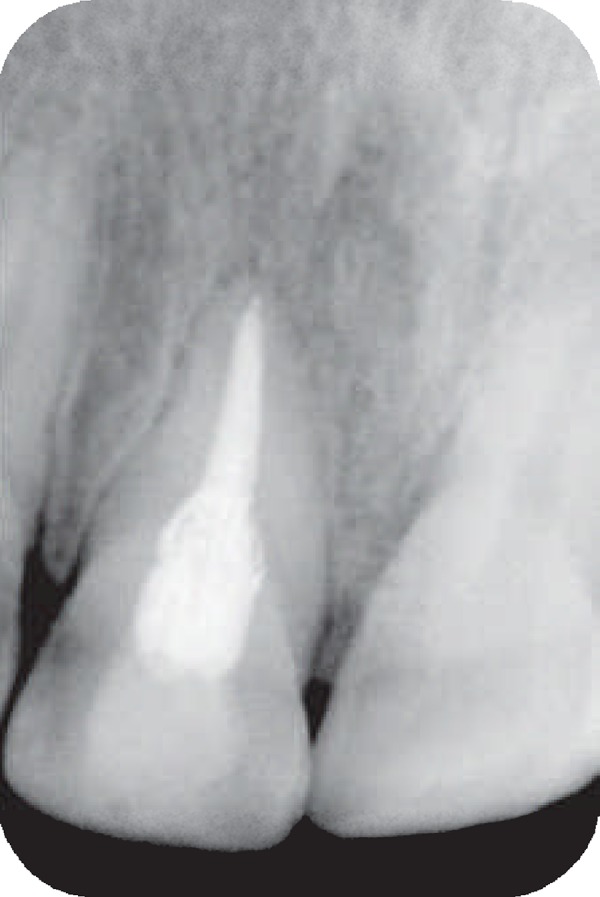
Healing of periapical lesion one year after root canal therapy

An eight-year-old girl attended our clinic because of trauma that caused complicated crown fractures in right maxillary central incisor. The patient referred to our office one week after accident. The patient's medical history was non- contributory. No spontaneous pain was reported by the patient and her main complaint was sensitivity to cold beverage in the fractured tooth. Clinical examination showed a complicated crown fracture with pulpal exposure on the right maxillary central incisor. Radiographic image (Figure 1) showed that the fractured tooth had immature apex. Under local anesthesia using 2% lidocanie and 1:80000 epinephrine (Darupakhsh, Tehran, Iran), and rubber dam placement cervical pulpotomy was performed in the tooth using a diamond bur (Diatech, Heerbrugg, Switzerland). The area was rinsed with 2.5% sodium hypochlorite (Golrang-Tehran, Iran). After that, the pulp was covered with pure CH powder (Merck, Darmstadt, Germany) that was mixed with physiologic saline (Samen Industries, Mashhad, Iran) to a very dry thick mix and which was condensed with a light, vertical pressure to a thickness of 3-4 mm. The tooth was restored with self cure composite resin (King Dental Corp., USA). Six months later the patient was followed up for clinical and radiographic evaluation. Radiographic images showed root development (Figure 2). After 10 years, however, the patient came back to the office because of sensitivity to percussion and palpation. A radiographic image (Figure 3) showed the presence of apical radiolucency around the right maxillary central incisor. The tooth did not respond to sensitivity tests such as cold, heat, and electric pulp tester. Other maxillary anterior teeth showed normal response to sensitivity tests. Under local anesthesia with 2% lidocaine and 1/80000 epinephrine and following isolation with rubber dam an access cavity was made and cleaning and shaping was performed using passive step back technique. Then the root canal was medicated with calcium hydroxide. After one week the calcium hydroxide was removed and the root canal was obturated with gutta- percha and AH26 root canal sealer. After one year patient was recalled. She had no clinical complain and complete healing was noticed in the tooth radiographic image (Figure 4).

## DISCUSSION

Vital pulp therapy is a reasonable treatment option particularly for the teeth with open apexes and vital pup (3). The pulp of the teeth that need vital pulp therapy may be mechanically or cariously exposed (7,8,10). Traumatic injuries such as complicated crown fracture produced mechanical pulp exposure. Complicated crown fractures usually permits pulp contamination by saliva and oral microorganisms. It has been stated that partial pulpotomy is the treatment of choice following pulp exposure of immature teeth with open apexes (5). In this case however, we did cervical pulpotomy because the late patient attendance for receiving dental treatment. The importance of coronal seal after pulp capping has been confirmed and acid-etch composite resin is introduced as the material of choice to cover capping material and provide reasonable esthetic for the patient particularly for the anterior teeth (12). In this case following pulp capping with CH, composite resin was employed as final restoration material.

Several materials have been introduced for pulp capping such as CH and MTA. Recent investigations have confirmed superiority of MTA in comparison to CH as pulp capping material in terms of inflammation and the thickness of calcified bridge beneath the capping material (13-15). However, MTA is a material that was marketed from 1998 and for this reason in this case we used CH as pulpotomy agent because at the time of treatment MTA was not available in the market (16).

There are disagreements among clinicians regarding the final treatment of immature teeth with open apexes that received vital pulp therapy (4,5). Some clinicians believe that there would be no need for further treatment as the closure of apical foramen is a sign of success (4). However, many others believe that the teeth may develop pulp canal space calcification following apex formation and therefore, root canal therapy should be performed to prevent total root canal obliteration (5). This case report showed that other than calcification, pulp necrosis and formation of periapical radiolucency may develop long time after initial vital pulp therapy. Previous investigations have stated that the presence of calcified bridge and development of root apex in immature tooth with open apexes is not a sign of complete success (17,18). This case report confirm that developing apical radiolucency and pulp necrosis may happen several years following initial treatment and for this reason we recommend long time follow-up for traumatized teeth even if they showed successful clinical and radiographic results at early recalls.
